# Prevalence and origin of prominent nutrient channels of the ilium bone on MR-imaging

**DOI:** 10.1007/s00256-025-04938-x

**Published:** 2025-05-29

**Authors:** Luca Albanese, Christoph Germann, Chantal Pauli, Dominic Gascho, Reto Sutter

**Affiliations:** 1 Department of Radiology, Balgrist University Hospital, University of Zurich, Forchstrasse 340, Zurich, Switzerland; 2 Systems Pathology and Functional Tumor Pathology, Zurich University Hospital, University of Zurich, Zurich, Switzerland; 3 Zurich Institute of Forensic Medicine, University of Zurich, Zurich, Switzerland

**Keywords:** Pelvic MRI, Nutrient vessels, Central vessel convolute

## Abstract

**Objective:**

Prominent nutrient vessels are commonly seen in the ilium bone, but little is known about their anatomical characteristics. The aim of this study was to investigate the frequency and morphology of these vessels and associated bone-marrow changes of the ilium using MRI.

**Materials and methods:**

MRI-examinations of the pelvis in 245 patients were analyzed retrospectively. Prominent nutrient vessels of the ilium were recorded, including vessel origin, anatomical characteristics such as branches, bone-marrow changes, and entry points into the bone.

**Results:**

Two hundred forty-five patients (54±16 years, range 18–88, 102 males) were included.

Prominent central nutrient vessels were found in virtually all patients on both sides of the ilium. All nutrient vessels arose from the iliolumbar artery, forming a breakthrough-anastomosis to the superior gluteal artery. Two branches were seen in 57.6% on the right and 61.2% on the left side, constituting the most prevalent branching pattern. Three branches were seen in a third for each side. One or four branches were seen in 3–4.5% for both sides. A prominent branching pattern we coined “central-vessel-convolute” (CVC) at the central part of the ilium was seen in 75% on either side. Perivascular fatty areas were found in 25% of cases, and in 3.7–2.4% adjacent bone-marrow edema was observed.

**Conclusion:**

Prominent nutrient vessels in the ilium are seen in almost all individuals, the majority exhibiting a specific CVC-pattern. These vessels may be surrounded by perivascular fatty areas; adjacent bone-marrow edema is rare. Recognizing the CVCs and the associated imaging findings should facilitate distinguishing normal anatomical structures from pathology.

**Supplementary Information:**

The online version contains supplementary material available at 10.1007/s00256-025-04938-x.

## Introduction

Magnetic resonance imaging (MRI) of the hip joint and pelvis has gained increased importance over the last decade and readily depicts abnormalities of the hip joint and the adjacent osseous structures [1–3]. In MRI examinations of the hip and pelvis often a network of prominent serpentine structures is present in the central portion of the ilium bone, occasionally surrounded by distinct lipomatous changes and bone marrow edema. In some cases, the diagnostic conundrum of these serpentine structures may lead to neoplastic differential diagnoses in a report, due to the presence of a highly vascularized mass-like structure in the ilium bone. We had several cases in our institution where the serpentine structures caused such a diagnostic conundrum, but we hypothesized that the prominent serpentine structures may actually be normal nutrient vessels.

So far, only a handful of scientific reports have described this anatomical region, most of which were conducted in the last century, included merely a few patients, and were only conducted with conventional radiographs or computed tomography (CT): these reports describe that central nutrient vessels in the ilium bone are normal variants and could be mistaken as a pathology, but the prevalence, the anatomical extent and morphology of these structures remains largely unknown and have not yet been further described in MRI imaging [4, 5]. At our institution, MRI of the hip and pelvis is commonly performed, e.g., in cases of suspected femoroacetabular impingement, hip abductor problems, or for pre-surgical planning. Even though the serpentine structures in the ilium bone are present in many of these examinations, they are seldom mentioned in the radiology report except in cases where pathologic findings are directly adjacent to the prominent serpentine structure.

The aim of this study was to investigate the frequency of prominent nutrient vessels in the central ilium bone and describe their morphologic patterns as well as the imaging characteristics of the adjacent bone.

## Materials and methods

### Patients

The study was approved by the institutional ethical committee (Kantonale Ethikkomission Zürich, KEK number: 2020 - 01140). The following inclusion criteria were applied: All patients participating have signed a general and informed consent for their participation in the study and the further use of relevant data for publication. Patients over 18 years who had undergone MRI of the pelvis between 2014 and 2020 with a minimum MR protocol of T1-, T2- and fluid sensitive sequences in the axial and coronal plane.

**Fig. 1:**
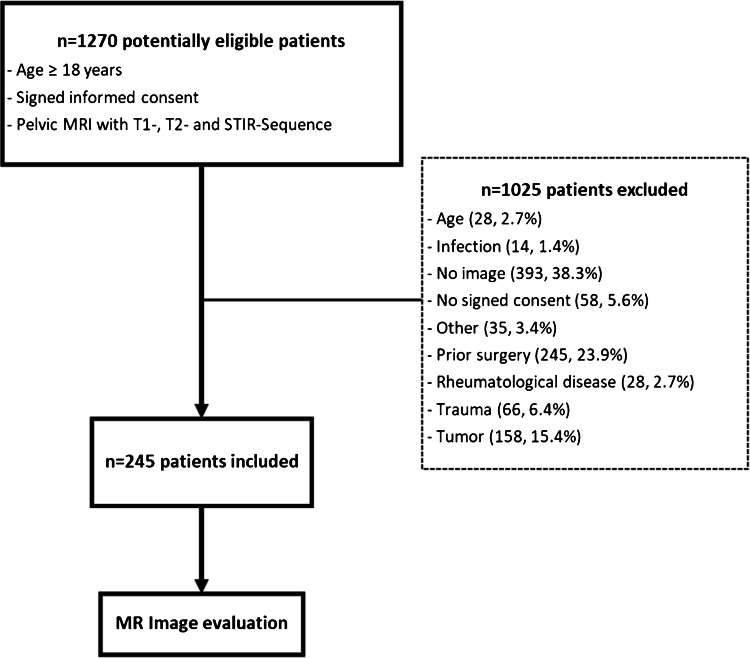
Flowchart for inclusion and exclusion criteria of patients with MR examinations of the pelvis. STIR: short tau inversion recovery

Exclusion criteria were the following: history of trauma or fractures, prior surgery (including prothesis and spondylodesis), cancer, infection or rheumatological diseases, or waiver of written informed consent. Our radiological database was searched for MRI examinations of the pelvis retrospectively in the given time frame. Of 1270 patients with MRI of the pelvis, 1025 patients were excluded, resulting in 245 remaining cases (Fig. 1).

### MR image analysis

Image analysis was performed on our institution’s picture archiving and communication system (PACS) (Merlin, Phoenix-PACS) and the imaging characteristics were recorded in an anonymized database (REDCap, 12.3.0, Vanderbilt University) by a postgraduate researcher. Due to the use of different scanners from different manufacturers and minor protocol adjustments made over time, detailed MRI protocol parameters are not provided here. However, in all cases, T1, T2 and fluid sensitive sequences were acquired.

### Nutrient vessels and central vessel convolutes (CVC)

**Fig. 2:**
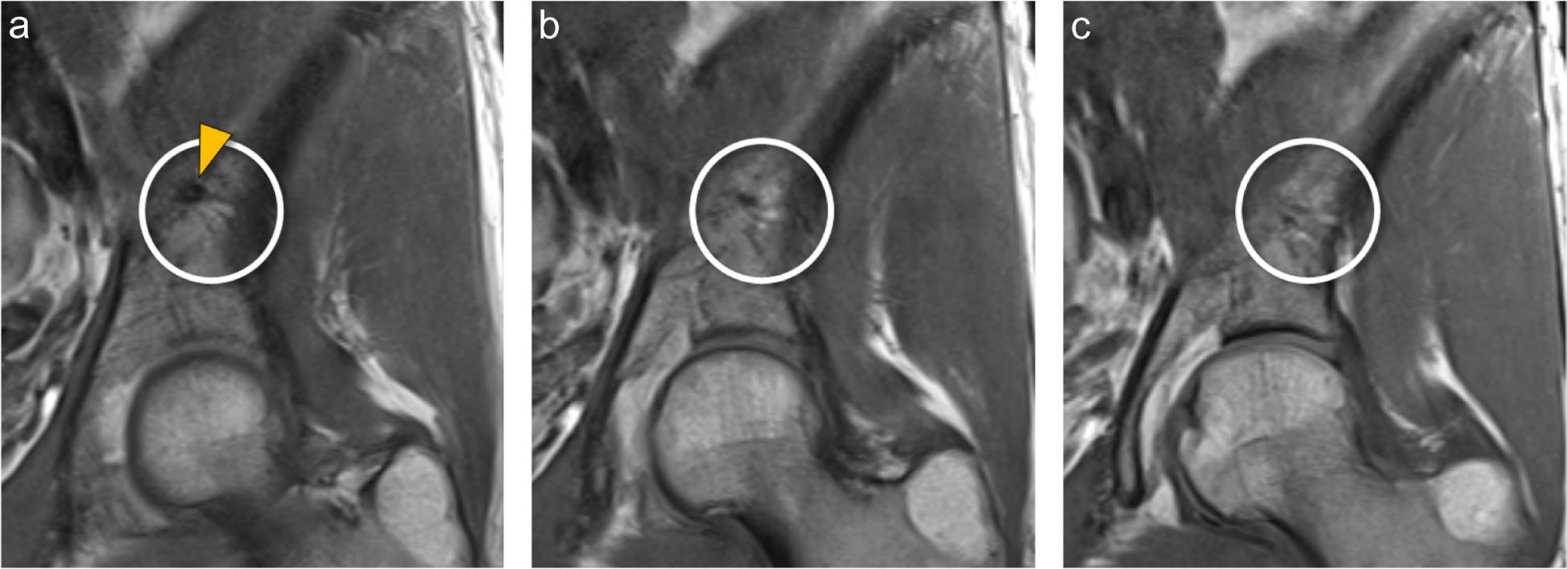
Central vessel convolute (CVC) in a 41-year-old female. Coronal T1-weighted MR images of the left ilium bone, shown from posterior to anterior. **a** Entry point of the iliolumbar artery (arrowhead) into the ilium bone. **b**, **c** The branches of the iliolumbar artery spread radially, forming the CVC (marked by the circle)

The presence of the nutrient vessels was assessed on the right and left ilium bone. The number of entry points of main vessels and branches was counted, and their trajectory through the bone was traced, including the presence of a breakthrough-anastomosis (i.e., anastomosis on the outer side of the ilium bone connecting with gluteal vessels). The central vessel convolute (CVC) was defined as a prominent network of small vessels in the center of the ilium bone originating from a nutrient vessel at its entry point at the medial side of the ilium bone (Fig. 2, Supplementary Fig. S3). The presence of CVC was assessed, and the area of the CVC was measured using the standard caliper tool of the PACS software.

### Entry points and nutrient vessel branches

**Fig. 3:**
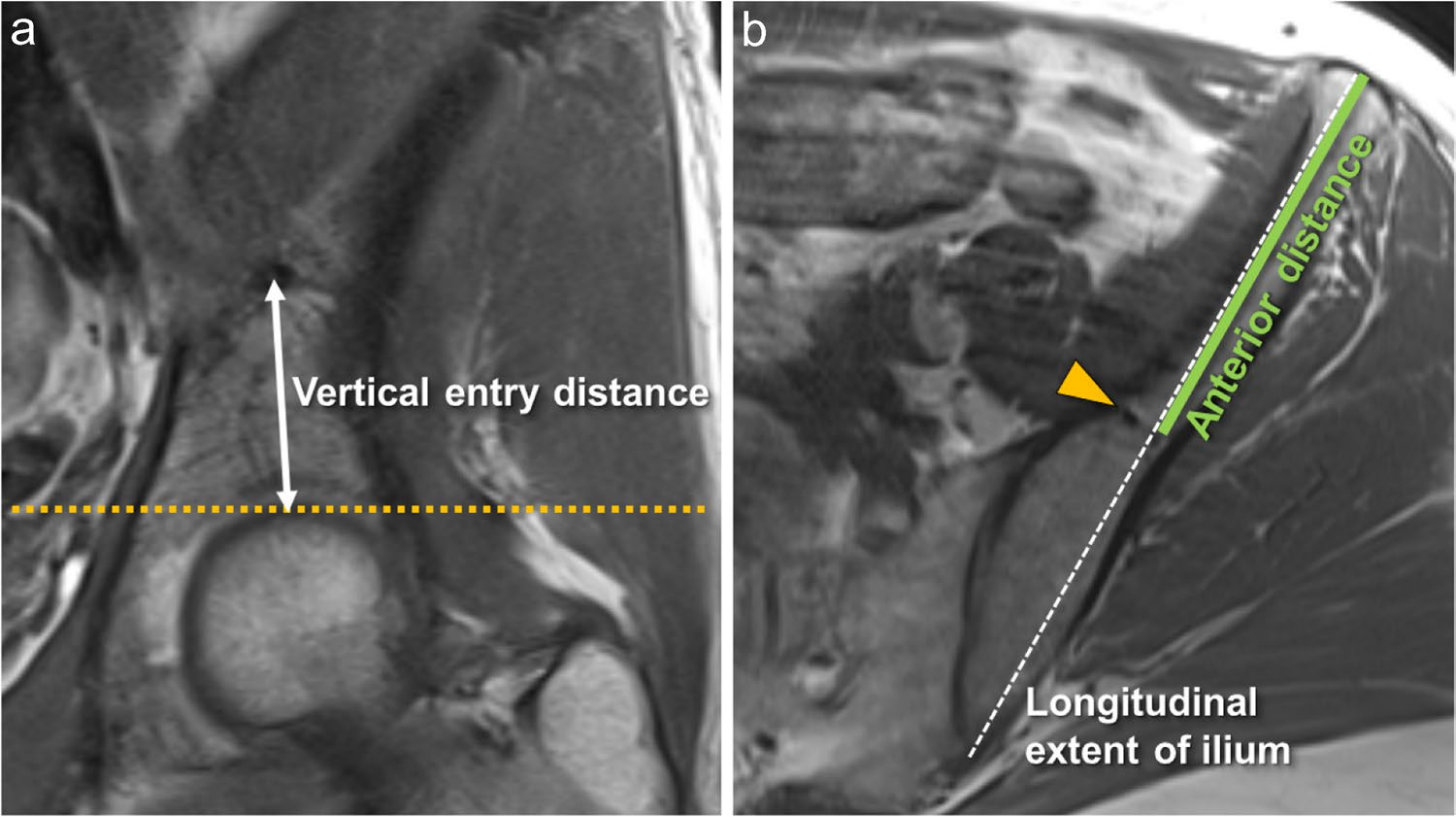
Location of the entry points of the nutrient vessel in a left ilium bone. **a** The vertical entry distance of the nutrient vessel was measured in the coronal plane from the most apical point of the femoral head (dotted yellow line) to the entry point of the vessel. **b** In the axial plane the longitudinal extent of the ilium bone is shown (dashed white line). Parallel to the longitudinal extent of the ilium the anterior distance from the vessel entry point (arrowhead) to the anterior superior iliac spine was measured (thick green line)

**Fig. 4:**
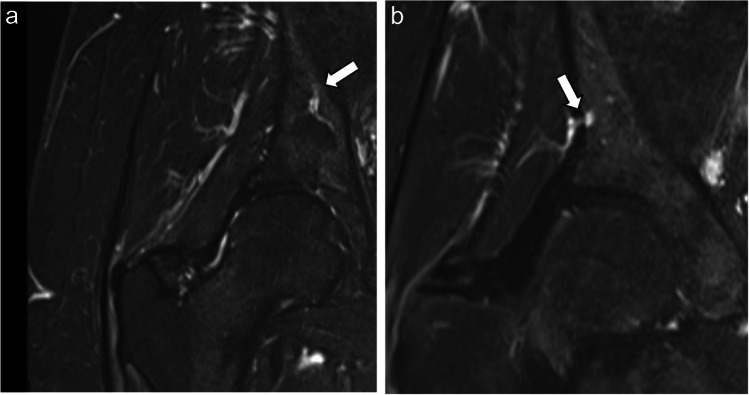
The entry and exit points of the nutrient vessel are shown on two STIR images of the right hip in a 56-year-old female patient. **a** The entry point of the nutrient vessel is seen at the medial side of the ilium bone (arrow). **b** The exit point (anastomosis with superior gluteal artery) can be seen in a more anteriorly located image at the lateral side of the ilium bone (arrow). STIR: short tau inversion recovery

In a sub-analysis, the exact location of vascular entry points and the three-dimensional distribution were measured in 150 cases on both sides. The location of the entry point of the nutrient vessel into the internal surface of the ilium bone was described using the distance from the most apical point of the femoral head, which we named “vertical entry distance” (Fig. 3). In the axial plane, three measurements were performed: Firstly, the longitudinal extent of the ilium bone was assessed measuring the distance between the anterior superior and posterior superior iliac spine. Secondly, from this three-dimensional longitudinal axis, the vessel entry point was measured on a perpendicular distance to the longitudinal axis as well as, thirdly, the distance of the crossing point to the anterior superior iliac spine (Fig. 3). The branching number of nutrient vessels, the branching convolute area, distribution area of either fatty signal or edematous signal were assessed in the axial and coronal plane. Additionally, the presence and location of exit points of the nutrient vessel branches on the outer surface of the ilium bone were described (Fig. 4).

### Bone marrow changes adjacent to the CVC

**Fig. 5:**
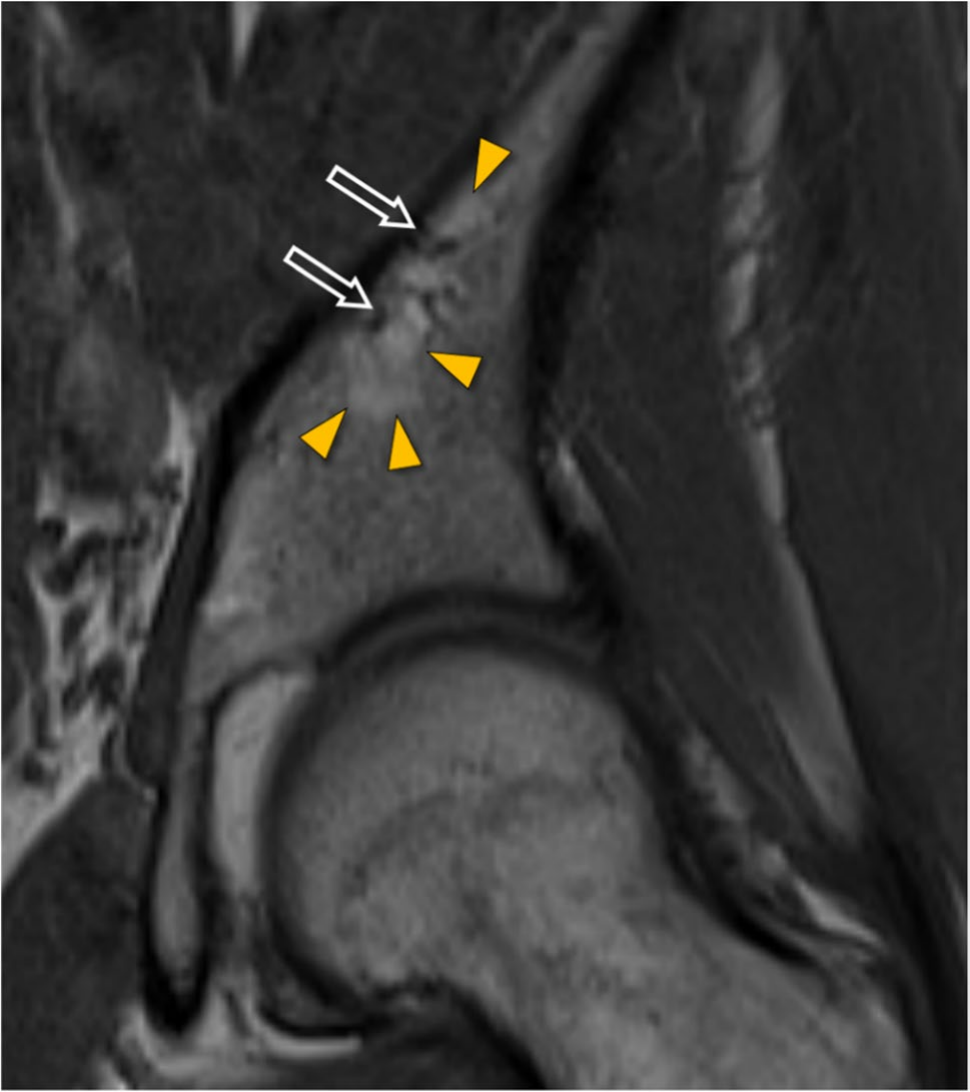
Central vessel convolute (CVC) with surrounding fat signal in a coronal T1-weighted MR image of the left pelvis in a 65-year-old female. The CVC with two main branches is indicated by outline arrows, and the adjacent area with fat signal is indicated by orange arrowheads

In the bone adjacent to the CVC, the presence of fatty signal alterations was recorded (defined as distinct fatty bone marrow on T1-weighted images directly adjacent to the CVC) and the presence of bone marrow edema on STIR-images was recorded. For both these entities, the area of the affected bone was measured (Fig. 5).

### Bone extraction in three cadaveric specimens

In addition to the retrospective MRI analysis, we performed a macroscopic and histological analysis of the central part of ilium bone in three cadavers to investigate the presence and confirm the histological nature of the prominent nutrient vessels (Supplementary data).

### Statistical analysis

Descriptive statistics (mean value ± standard deviation) was applied to assess the prevalence and distribution of nutrient vessels of the ilium bone as well as the prevalence of vessel convolutes and the presence of either a fatty signal or bone marrow edema. The difference between males and females in respect to branches and vessel alterations (CVC, fat signal or edema) was assessed with the unpaired Mann-Whitney-*U*-test, with *p*= 0.05 indicating statistical significance. Correlation between sides in respect to branches and vessel pattern was determined with the Wilcoxon-signed-rank-test. Interobserver agreement was calculated with Cohen’s kappa for the presence or absence of nutrient vessels and vessel convolutes. All statistical analyses were performed with dedicated software (SPSS, version 28.0.1.0, IBM).

## Results

The study population comprised 102 male patients (41.6%) and 143 female patients (58.4%), with a mean age of 54 years (± 16), and an age range from 18 to 88 years.

### Nutrient vessels and central vessel convolutes (CVC)

Prominent nutrient vessels with a clear entry point/nutrient foramen were present in 99.2% on the right side (243/245) and 100% on the left side, all originating from the iliolumbar artery. On both sides there were two cases (0.8%) where two distinct osseous entry points could be determined. In 74.1% a CVC was present on the right side, and 74.3% on the left side (Table 1). There was no statistically significant difference for the number of CVC for either side (*p*= 0.808) or gender (*p*= 0.576). There was a perfect interreader agreement for the presence of nutrient vessels (ĸ= 1) and a substantial interreader agreement for the presence of CVC (ĸ= 0.72–0.79).

**Table 1:** Assessment of a central vessel convolute (CVC) and bone marrow changes in the ilium bone. For the number of findings, the absolute numbers and their relative percentages are given. The p-value reflects the relation to the presence of the changes and the area distribution compared between the left and right side

	Right Side						Left Side						
	Number of nutrient vessels: 243						Number of nutrient vessels: 245						
	Number of findings		Mean value (cm^2^)	SD (cm^2^)	Min (cm^2^)	Max (cm^2^)	Number of findings		Mean value (cm^2^)	SD (cm^2^)	Min (cm^2^)	Max (cm^2^)	*p* -value (Wilcoxon)
Presence of CVC in the ilium bone													
CVC	180	74.1%	1.33	0.60	0.345	3.64	182	74.3%	1.33	0.73	0.39	5.96	0.892
*No CVC*	*63*	*25.9%*	*-*	*-*	*-*	*-*	*63*	*25.7%*	*-*	*-*	*-*	*-*	*-*
Bone marrow changes adjacent to the CVC													
Fat signal	62	25.5%	1.24	0.47	5.47	0.47	65	26.5%	2.81	1.17	0.72	5.67	0.277
Edema	9	3.7%	2.21	1.18	0.43	4.38	6	2.4%	2.81	1.54	5.20	1.15	0.273

The area covered by the CVC was 1.33 cm^2^ ± 0.60 cm^2^ (standard deviation) for the right side and 1.33 cm^2^ ± 0.73 cm^2^ for the left side, without a statistically significant difference between the right and left side (*p*= 0.892) (Table 1).

### Nutrient vessel branches

After entry into the bone, the nutrient vessels branched off in the large majority of cases: on the right side, in 4.1% the vessel remained as a single vessel. In 57.6% of cases there were 2 branches with one branch continuing along a perpendicular path through the acetabular portion of the bone and the other branch moving along the longitudinal axis of the ilium bone to a cranial direction. In 33.7% of cases, 3 branches were visible and in 4.5% of cases 4 distinct branches were seen. On the left side, in 4.1% of the cases the entry vessel remained as a single vessel. In 61.2%, 2 branches were seen with identical distribution (one perpendicular, one longitudinal). In 31.0%, there were 3 and in 3.7% 4 distinct branches, showing a very similar distribution for both sides of the ilium. There was no statistically significant difference between the number of branches compared to either side of the ilium (*p*= 0.345) or the number of branches compared by gender (*p*= 0.924).

**Fig. 6:**
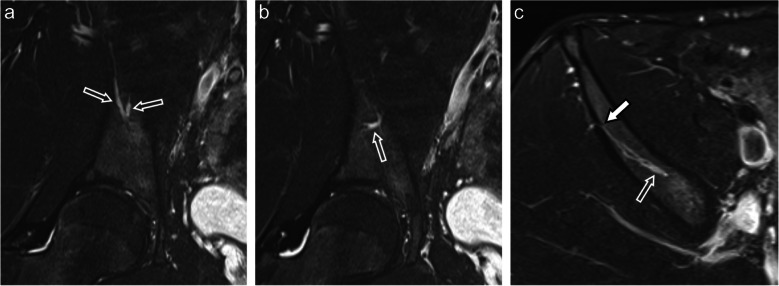
MRI of the right ilium bone in a 25-year-old male showing the peripheral branches of a CVC. **a**, **b** Several prominent branches (outline arrows) can be seen in two adjacent coronal STIR images. **c** On the axial STIR image two branches of the CVC show an almost parallel course (outline arrow), and the exit point of anterior branch (white arrow) can be seen on the lateral surface of the ilium bone, forming an anastomosis with the superior gluteal artery. STIR: short tau inversion recovery

In most cases, the nutrient vessels broke through the lateral surface of the ilium bone, forming an anastomosis with a branch from the superior gluteal artery. Such an osseous breakthrough of either one or multiple main branches was seen in 98.8% of cases on the right side and 97.6% on the left side (Fig. 6).

### Location of entry points

For the vertical entry distance on the right side, a mean distance of 36.6 mm (± 5.6 mm standard deviation) was measured, for the left side, a mean distance of 36.8 mm (± 6.4 mm) was observed, showing similar measurement results for both sides (*p*= 0.488).

In the axial plane, the longitudinal extent of the ilium bone measured 156.4 mm (± 8.7 mm) for the right side and 156.8 mm (± 8.5 mm) for the left, with no statistical relevant difference when comparing mean values to sides (*p*= 0.963).

The anterior distance to the ASIS (anterior superior iliac spine) was 75 mm (± 6.1 mm) for the right and 74.1 mm (± 5.7 mm) for the left side (*p*= 0.046). The entry point coefficient was 0.484 (± 0.029) for the right and 0.479 (± 0.029) for the left side, meaning that the vessel entry point from the internal side into the ilium bone would be almost exactly in the middle of the longitudinal axis of the ilium bone using ASIS and PSIS as reference points (Table 2).

**Table 2:** Location of vessel entry points into the ilium bone. The vertical distance of the entry point from the apex of the femoral head was assessed in the coronal plane. The longitudinal extent of the ilium bone and the anterior distance to the anterior superior iliac spine (ASIS) were assessed in the axial plane. The “longitudinal coefficient” is calculated from the two measurements in the axial plane (vessel entry divided by the length of the longitudinal axis of the ilium bone). The result was a coefficient close to 0.5, meaning that the vessel entry point was approximately in the middle of the ilium bone

	Right Side				Left Side				
Location of vessel entry points	Mean value	SD	Min	Max	Mean value	SD	Min	Max	*p* -value (Wilcoxon)
Coronal plane									
Vertical entry distance	36.6 mm	5.6 mm	26.7 mm	57.1 mm	36.8 mm	6.4 mm	14 mm	56.9 mm	0.488
Axial plane									
Longitudinal extent ilium bone	156.4 mm	8.7 mm	140.0 mm	179.6 mm	156.8 mm	8.5 mm	139.2 mm	182.4 mm	0.963
Vessel entry: Anterior distance to ASIS	75 mm	6.1 mm	55.1 mm	89.8 mm	74.1 mm	5.7 mm	56.5 mm	88.4 mm	0.046
*Vessel entry: Longitudinal coefficient*	*0.484*	*0.029*	*0.375*	*0.556*	*0.479*	*0.029*	*0.361*	*0.547*	*0.03*

### Bone marrow changes adjacent to the CVC

In 25.5% of ilium bones (*n*= 62) a prominent T1-weighted fat signal was detected around the CVC for the right side, and in 26.5% (*n*= 65) for the left side. Relative to the number of patients with a CVC, on the right side the fat signal was detected in 34% of CVC (62 cases of 180 CVC), and on the left side it was detected in 36% of CVC (65 cases of 182 CVC), respectively. The area of this prominent fatty signal was 2.47 cm^2^ ± 1.25 cm^2^ on the right side and 2.81 cm^2^ ± 1.17 cm^2^ on the left side, without statistically significant differences between the sides (*p*= 0.277). Also, regarding the number of prominent fatty signals there was no statistically relevant difference for either side or gender (*p*= 0.564 vs. *p*= 0.662).

An edematous signal alteration centered on the CVC was seen in 3.7% (9 cases) on the right and 2.4% (6 cases) on the left side. The area of the edematous signal alteration was 2.17 cm^2^ ± 1.18 cm^2^ for the right side and 2.43 cm^2^ ± 1.54 cm^2^ for the left side, without a statistically significant difference regarding sides (*p*= 0.273). The occurrence of edematous signal alterations was not statistically different regarding side or gender (*p*= 0.257 vs. *p*= 0.358). There were no statistically significant differences regarding the number of patients with fat signal alterations, and the number of patients with edema adjacent to the CVC for either gender (*p*= 0.662, and 0.358, respectively), or side (*p*= 0.564, and 0.257, respectively).

### Bone analysis in three cadaveric specimens

In the ilium bone of the three cadaveric specimens, the presence of the prominent nutrient channels was macroscopically visible in all specimens (Supplementary Fig. S1). The histology confirmed that the CVC in the ilium bone were small arteries (Supplementary Fig. S2).

## Discussion

Our study shows that nutrient vessels of the ilium bone are very frequently present on MRI examinations (> 99%), and they are most frequently visualized with a CVC branching pattern (in about three quarters of individuals). The existence of nutrient vessels in the ilium bone has been known since the last century and their occurrence has been described using conventional radiographs, computed tomography as well as anatomical cadaver studies [4–7]; a main focus of prior studies was on the role of nutrient channels in fetal and infant ilium bones concerning the initiation of enchondral ossification and bony development [8, 9].

The nutrient arteries in the ilium arise primarily from the iliolumbar artery and to a lesser extent from the obturator artery (both arising from the internal iliac artery) and have their entry point at the cephalad side of the medial surface of the ilium anterior to the sacroiliac joint and form, after invasion and branching within the bone, an anastomosis with the superior gluteal artery (from the external iliac artery) on the gluteal/external surface of the ilium bone [4, 6, 10, 11]. The first description of the location of the nutrient foramen was done by Ebraheim et al. in 15 cadaveric specimens, using the distance of the nutrient foramen to the sacroiliac joint and the pelvic brim [6]. The study stated that the nutrient vessels and entry points were “constant” in appearance and location. Interestingly, the ilium bone does not only have a foramen where the nutrient artery is entering the cortical bone, but the underlying trabecular bone also features an altered morphology, with a distinct osseous collar accompanying the proximal part of the artery [7].

A branching in two main directions has been reported in the acetabular portion and the longitudinal iliac portion of the ilium [4, 12], coinciding with our own findings. The incidence of such nutrient vessels in the ilium has been estimated to be at 80% in the literature, with the right side being both slightly more prevalent (in the male gender) and slightly larger in dimension [4, 5]. Our findings show a near 100% incidence for the presence of nutrient vessels, without a side or gender preference.

Most studies on nutrient vessels were performed in the last century and were focused on the possibility of traumatic fractures or a possible increased risk of stress fractures due to the cortical and trabecular disruption by the presence of nutrient vessels, and also the risk of iatrogenic injury to the vessels has been discussed [6, 11, 14, 17–20, 13, 15]. The presence and the precise locations of vascular channels and nutrient arteries have been described in depth for other anatomical areas, for example in the femur, tibia, and fibula [16, 21]. In particular, the risk of iatrogenic damage to nutrient vessels has been thoroughly demonstrated in the example of the tibial nutrient artery in cases of external surgical fixation after trauma to limit blood loss or compromising fracture healing, putting the existence of nutrient vessels into clear clinical perspective [16, 23, 22, 24].

In our study, we demonstrated the presence of nutrient vessels in the ilium, their relatively constant pattern and provide a detailed description of the location and trajectory through the bone. As was suggested by Ebraheim et al. care should be taken when planning surgical interventions of the ilium bone, so as not to injure the nutrient vessels during osteosynthesis, cancellous bone graft harvest or bone marrow biopsies [6].

The mere presence of such nutrient channels may easily be confused with a pathological finding, such as a fracture or a neoplastic process on conventional radiography or CT [4, 19, 26, 27, 25, 28]. The confusion with fracture lines is an obvious risk, given the radiolucency in conventional radiographs and the cortical and trabecular disruption in both radiography and CT. The possibility of confusing nutrient vessels or nutrient channels with neoplastic processes was described by Coffre et al. in the example of CT evaluation of osteosarcoma of the tibia, fibula and humerus, arguing that in any unclear case, comparison with the contralateral side is warranted [29]. The same considerations are true for the ilium bone, and the data about the location and morphological features of the nutrient vessels and the CVC presented in our study can be helpful to identify the normal anatomical findings, and thus avoid confusion with pathologies of the ilium bone. This is of special importance because usually an increased number of blood vessels is seen as a sign of malignancy [30], which is clearly not the case for the CVC, which was seen in the majority of our patient population.

The comparison of pelvic MRI examinations in our study showed a vessel entry from the iliolumbar artery into the ilium bone being present in almost all cases (99.2% for either side), and where a vessel was detected, a breakthrough anastomosis with the superior gluteal artery was seen. The location of entry points into the ilium bone, as well as the intraosseous course and exit point was constant and showed little variability among gender and age. Furthermore, in the histological analysis included as Supplementary data, we were able to confirm that the prominent vessels in the central part of the ilium bone are indeed nutrient arteries.

At MRI the distinct morphological appearance of a CVC with its serpentine branching pattern of small vessels was seen in 71–74% of ilium bones in our study. In about a third of these cases with CVC, a prominent fat signal on T1-weighted images was seen next to the vessels of the CVC and in the adjacent central part of the ilium bone. The fat signal around the CVC had a diffuse appearance, unlike in cases of intraosseous lipoma, which are more circumscribed and feature a small transition zone [31]. It might be hypothesized, however, that the presence of vascular pulsations by the CVC contributes to the development of the fatty areas at this location, similar to one of the hypotheses for the development of intraosseous lipomas due to a vascular stimulus [32].

In 15 cases, an edematous signal was seen adjacent to the CVC on fluid-sensitive sequences. These edematous signal alterations had a more variable morphology and extent than the fatty marrow changes, but these are the cases most easily misidentified as neoplastic lesions, which also commonly present with hyperintense signal in the fluid-sensitive sequences. The findings for vascular branching, CVC and perivascular bone marrow changes showed identical distributions for both sides and both genders.

Our study describes the presence and location of the nutrient vessels of the ilium bone and introduces the frequent and prominent vascular pattern of the CVC, which was seen in three quarters of all patients. While commonly the increased number of blood vessels is a sign of a neoplasm, in this specific location it should be considered as a normal finding. Even if adjacent signal alterations of the bone marrow are present in the ilium bone, this should not be confused with pathological findings such as a fracture or a neoplastic process.

## Supplementary Information

Below is the link to the electronic supplementary material.

ESM 1 (DOCX 349 KB)

## Data Availability

All patient relevant data is stored at the hospital site. Clinically relevant data is stored within the KISIM system, images were obtained from the hospitals picture archiving and communication system (PACS) Merlin, Phoenix PACS system. Data entry, and statistical analysis was performed using REDCap, (V.12.3.0, Vanderbilt University).

## Abbreviations

ASIS: anterior superior iliac spine
CVC: central vessel convolute
STIR: short tau inversion recovery
