# Supplementary material for: Prevalence and origin of prominent nutrient channels of the ilium bone on MR-imaging
Source: Skeletal Radiol. 2025 May 29;54(10):2127–35. doi: 10.1007/s00256-025-04938-x (PMC12361307; doi:10.1007/s00256-025-04938-x)
Supplement: Supplementary file 1 — (DOCX 349 KB) [file 256_2025_4938_MOESM1_ESM.docx]

# Supplementary data

**Bone extraction in three cadaveric specimens**

In addition to the retrospective MR analysis, we performed a macroscopic and histological analysis of the central part of ilium bone in three cadavers to investigate the presence and confirm the histological nature of the prominent nutrient vessels. For this purpose, the central part of the ilium bone was extracted in three cadavers at the institute of forensic medicine, after the institutional review board approved the extraction of the bone.

**Materials and Methods for Histology:** The ilium bone tissue was fixed in 4% buffered formalin for 72 hours. Upon identification of the vessel entry point/nutrient foramen cross sections were made at 4 mm thickness by an EXACT 312 pathology saw. Selected bone slices were embedded in paraffin and 2 μm sections were cut. Hematoxylin and eosin (H&E) as well as Elastica van Gieson (EvG) stainings were used for histology.

**Results:** In the ilium bone of the three cadaveric specimens, the vessels and the adjacent bone marrow were macroscopically visualized and the presence of the prominent nutrient channels was confirmed in all specimens (Supplementary Fig. S1). The histological evaluation showed that the composition of the central vessel is compatible with that of arteries, with a distinctively rounded, non-collapsed lumen and multiple layers of elastic fibers in a prominent tunica media, intertwined with smooth muscle fibers, as well as the presence of multiple smaller arterioles and capillaries in the tunica adventitia (i.e. vasa vasorum) (Supplementary Fig. S2). Histological assessment showed no signs of pathology in the juxtavascular area.


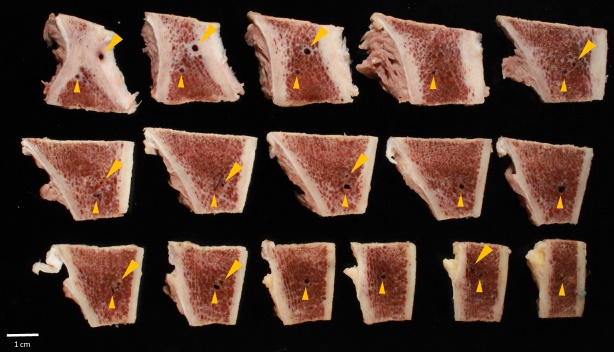


**Supplementary Figure S1**: Macroscopic photographs of sequential cross sections of the central portion of the right ilium bone extracted
from the cadaver of a 23-year-old man. Orange arrowheads indicate nutrient vessels and vessel branches.


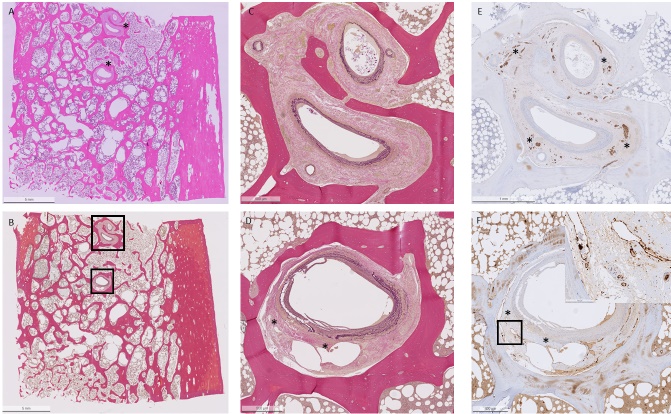


**Supplementary Figure S2:** Histological examination of the ilium bone from the cadaver shown in supplementary figure S1. A) At histology the arteries (asterisks) are visible in a hematoxylin and eosin staining. B-D) Elastica van Gieson staining is illustrating elastic fibres in the arteries (the two boxes in B mark the location of the images at higher magnification in C and D. In the loose connective tissue around the arteries thin walled capillaries (asterisks in D) are embedded. E) S100 staining in brown is showing small peripheral nerves embedded in the loose connective tissue (asterisks). F) Thin walled capillaries (asterisks) are detected in brown with a CD31 staining; the box in F shows the location of the insert image.


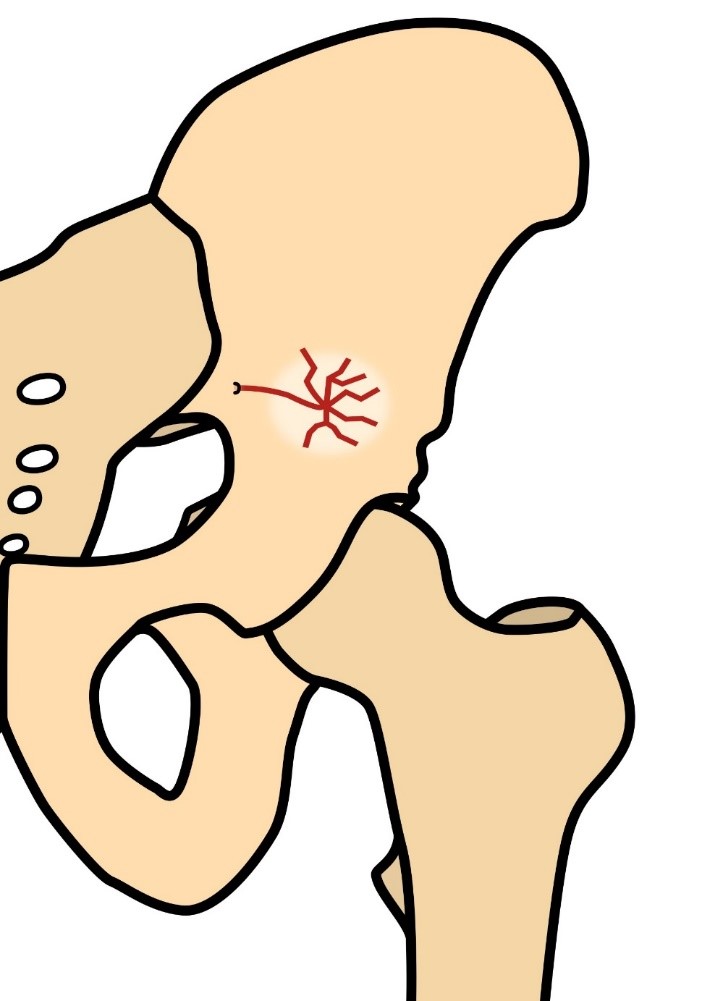


**Supplementary Figure S3**: Schematic depiction of the central vessel convolute (CVC) with an entry vessel and the distinct serpentine distribution of the smaller vessels. Implied is the perivascular fatty marrow change in lighter coloration.
